# Challenges to conducting epidemiology research in chronic conflict areas: examples from PURE- Palestine

**DOI:** 10.1186/s13031-016-0101-x

**Published:** 2017-02-22

**Authors:** Rasha Khatib, Rita Giacaman, Umaiyeh Khammash, Salim Yusuf

**Affiliations:** 1 Department of Public Health Sciences, Loyola Medical Center, Maywood, IL USA; 2 Institute of Community and Public Health, Birzeit University, Birzeit, occupied Palestinian territory Palestine; 3 UN Relief and Works Agency for Palestine Refugees in the Near East, East Jerusalem, occupied Palestinian territory Palestine; 4 Population Health Research Institute, McMaster University and Hamilton Health Sciences, Hamilton, Canada

**Keywords:** Low- and middle- income countries, Epidemiology, Conflict zone, Cardiovascular disease, Occupied Palestinian territory

## Abstract

Little has been written on the challenges of conducting research in regions or countries with chronic conflict and strife. In this paper we share our experiences in conducting a population based study of chronic diseases in the occupied Palestinian territory and describe the challenges faced, some of which were unique to a conflict zone area, while others were common to low- and middle- income countries. After a short description of the situation in the occupied Palestinian territory at the time of data collection, and a brief overview of the design of the study, the challenges encountered in working within a fragmented health care system are discussed. These challenges include difficulties in planning for data collection in a fragmented healthcare system, standardizing data collection when resources are limited, working in communities with access restricted by the military, and considerations related to the study setting. Ways of overcoming these challenges are discussed. Conducting epidemiological research can be very difficult in some parts of our turbulent world, but data collected from such regions may contrast with those solely from politically and economically more stable regions. Therefore, special efforts to collect epidemiologic data from regions engulfed by strife, while challenging are essential.

## Introduction

Conducting population based studies in low- and middle- income countries can be challenging due to poor infrastructure and limited resources. Such efforts can be even more challenging if the communities are in regions of war and strife. This paper describes issues encountered in designing and conducting the Prospective Urban Rural Epidemiology (PURE) study in the occupied Palestinian territory (oPt). In addition to the limited resources faced by other low and middle-income countries, research in the oPt is compounded by additional challenges related to its political situation and the military occupation. This paper provides a brief background on the current situation in the oPt followed by a description of PURE, in order to provide context to understand the challenges faced. Specific methods or results are not presented here; instead the aim is to describe the challenges encountered and how they were addressed during the data collection period of the baseline phase of PURE in the oPt. Our experiences could be useful to other researchers conducting epidemiological research in challenging and constrained settings.

## oPt in context

The limited data from the oPt suggests an epidemiologic transition, where the leading causes of death have changed from infectious to chronic diseases [1]. The leading causes of death are heart disease, constituting 26 % of deaths, cerebrovascular disease (12 %), and cancers constituting 11 % of all deaths [2]. Epidemiology data on cardiovascular disease and cancer are scarce, and estimates are based on routine data gathered by the Ministry of Health and from national surveys conducted by the Palestinian Central Bureau of Statistics. Reliable data on risk factors, treatments, and outcomes of cardiovascular diseases are limited. The available information is based on surveys conducted using self-reported data [1]. Higher quality data are limited and based on small studies which are not representative [3, 4].

The unique political situation in the oPt warrants special attention and requires that a number of factors that are usually not part of epidemiologic investigations of chronic diseases should be studied in trying to understand the causes, prevention and treatment of common diseases. The long term chronic conflict has increased exposure to violence, adding stressors that could heighten the impact of stress on cardiovascular diseases and also on health behaviors (eg. smoking, diet, physical activity). This conflict has also impoverished individuals and communities, with limited resources for health care [5–7]. Restrictions in travel between the West Bank and Gaza Strip, and also between communities within the West Bank compound lifestyle behaviors, resulting in regional differences in lifestyles which affect cardiovascular diseases.

The oPt has one of the largest refugee populations in the world which influences living conditions, socioeconomic status, and delivery of health care. Palestinians became refugees after the establishment of the state of Israel in 1948, and about 4.5 million refugees and their descendants are registered by the United Nations Relief and Works Agency for Palestine Refugees in the Near East (UNRWA). Almost a third of Palestinian refugees still live in camps inside and outside the oPt, although these camps are now urban settlements, not tents [5]. Palestinian refugees have been living in these camps for over 60 years, and their entire life experience is influenced by the special circumstances that they have experienced.

In addition, certain characteristics of the healthcare system which influence screening, prevention and management of disease. There are currently four different health care providers in the oPt: The Palestinian Ministry of Health (MoH), UNRWA, nongovernmental organizations (NGOs), and the private sector. Secondary and tertiary care is provided mainly through the MoH. Primary care is more fragmented: cities and most villages receive care from MoH, a few villages receive care from NGOs, and refuges receive care from UNRWA. The private sector provides primary as well as secondary care, yet it is not well regulated by a supervisory body [1]. It is thus important to assess the impact on disease outcomes across the different health care providers, as availability and quality of care may vary.

The unique circumstances of this population and its context adds to the importance of collecting local data with a large enough population to inform policies for chronic disease prevention and programs for their management. So far the effects of the long term chronic conflict have been studied mainly as they pertain to mental health and overall wellbeing [6–8]. Collecting longitudinal data on outcomes of common diseases can improve the understanding of the effects of the long chronic conflict on chronic diseases. Development and implementation of policies at the local level are necessary for designing prevention programs in order to control common diseases. Such programs require high quality data drawn from a large sample representing the entire population.

## PURE overview

PURE is a prospective cohort study designed to collect data on social, environmental, behavioral, biological, and genetic factors that contribute to the development of cardiovascular diseases in high-, middle- and low- income countries [9]. This study provides a simple design to be used in countries with limited resources for research, keeping in mind the importance of ensuring the collection of high quality data. Standardized forms are used to collect data at the community level, household level, and individual level with the aim of understanding how risk factors at these different levels may be associated with cardiovascular disease. Once the baseline data collection of the cohort is completed in each country, regular follow up visits are planned at three year intervals to follow up study participants for clinical events.

**Fig. 1:**
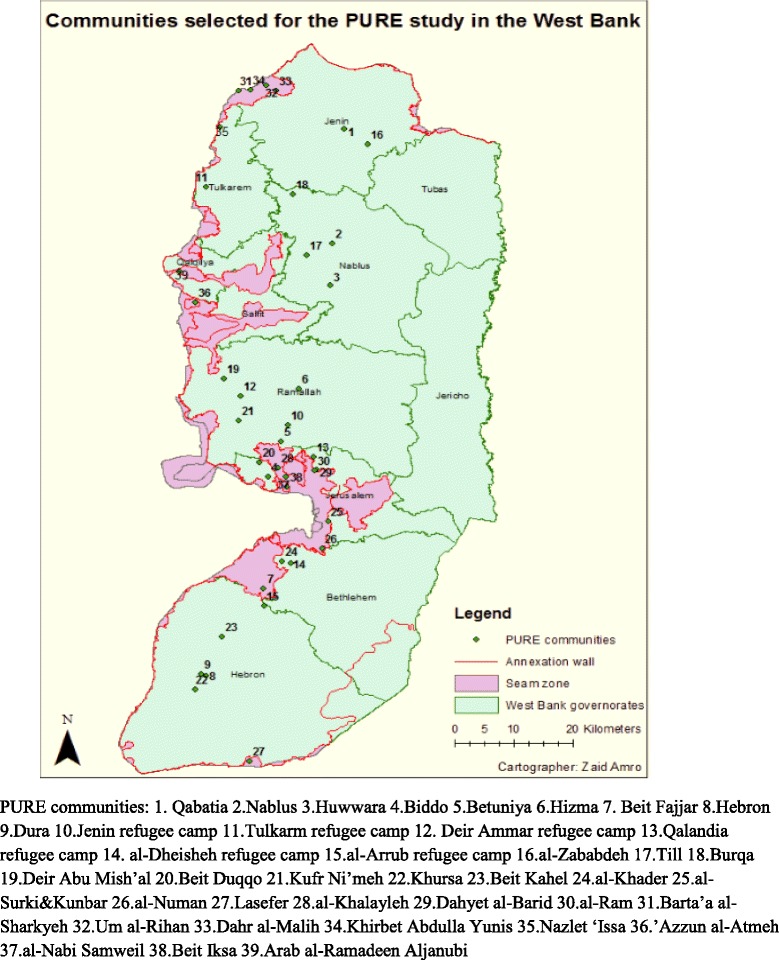
Map of the West Bank indicating communities included in PURE Palestine

For PURE Palestine, plans for data collection initially included 48 communities in the West Bank and Gaza Strip. Due to the political unrest in the Gaza Strip at that time (6 day Israeli war on Gaza-November, 2012) the research team was unable to enter into the Gaza Strip and decided at the time, to focus data collection in the West Bank only. Data were collected from ten urban, nine rural, six refugee camp, and 15 seam zone communities in the West Bank (Fig. 1). Seam zone areas are mostly rural and can be defined as Palestinian communities located between the separation wall erected by Israel inside West Bank Palestinian land and the Green Line, that is, the official and internationally recognized borders between Israel and the West Bank [10].

Data were collected from households representing each selected community that have at least one family member between the ages of 35 and 70 years. Trained fieldworkers used standardized forms to collect information on household socioeconomic status, details on chronic diseases, medication intake, and chronic disease risk factors including smoking, physical activity, nutrition, and family history. Each community was then visited by a medical team (a trained nurse and lab technician) who collected anthropometric measures, resting blood pressure, spirometry measures, grip strength, and blood and urine samples.

## Challenges faced during PURE data collection

Research in low- and middle- income countries faces many challenges pertaining to limited resources and lack of research and medical infrastructure [11]. Conflict areas raise more challenges in terms of security and movement restriction difficulties, in addition to resource allocation to humanitarian acute response research as opposed research with more sustainable goals. While collecting data for PURE in the oPt we were faced with common challenges in such settings, in addition to a number of obstacles that were specific to the long-term chronic conflict in that region of the world. These challenges are presented along with how they were overcome by the research team:

## Working in a fragmented health care system

### Problem

The multiple health care sectors in the oPt (MoH, UNRWA, NGOs and private) pose specific challenges for research. Though the PURE sample was population based, a clinic setting with trained personnel was needed in each community to collect physical measures, blood, and urine samples from study participants. Primary health care is provided by different sectors depending on the location. For example, primary care clinics located in rural communities are managed by the MoH or by NGOs, whereas primary care clinics located in refugee camps are managed by UNRWA. Involving all stakeholders would likely compromise standardization of data collection. Further, coordinating with officials as well as staff from all stakeholders would not have been practical.

### Solution

To overcome these issues we decided to work with one stakeholder only. UNRWA had shown interest in research, specifically for cardiovascular disease prevention [12]. It was expected that participants from non-refugee communities would not be willing to attend clinics in refugee camps (where UNRWA clinics are located) due to the distance they have to travel and because refugee camp clinics are known to be overcrowded. Furthermore, it was unethical to take away resources from those in need from within the refugee camp community for study purposes. To overcome this problem, mobile clinics were set up, and trained nurses and lab technicians travelled to non-refugee community included in the study. Mobile clinics were set in place after contacting community leaders and municipalities. The support received from these leaders increased response rate as participants were more trusting knowing that this activity has been organized from within their community.

## Standardizing data collection

### Problem

Standardized data collection is important for cross-country as well as within-country comparisons. Due to access restrictions and unexpected closures in the oPt it was not possible to centralize training for the fieldworkers and medical teams.

### Solution

Training sessions were held to explain selection of households and household members’ strategy and to ensure that the forms were completed accurately. Nurses and lab technicians were also trained to complete the physical measures in a standardized manner. These sessions were held at three different locations, for the teams in the North, Center, and South of the West Bank. Once data collection started the research team visited each study site at least once to ensure adherence to study protocol. Team work and collaboration between fieldworkers and nurses was crucial. The fieldworkers were usually either from the same community or spent a longer time in the community and became familiar with community members. The nurses were less familiar with the community and the participants. Fieldworkers facilitated the nurses’ work by finding a location for the mobile clinic and also by contacting participants for their appointments and following up with them when they missed their appointment.

## Access restrictions

### Problem

Israeli checkpoints and road blocks, the separation wall, and military presence in the West Bank restricted movement and limited access of patients to health care facilities [13]. Therefore, movement restrictions in the West Bank were a foreseen challenge to this study. Data were collected from 39 communities in the West Bank (Fig. 1). Twenty-four of these communities were located within the separation wall with no major restrictions to access. However, at the time of data collection, the main entrances to three of these communities were blocked (Hizma, Biddu, and Beit Duqqu communities). The only way into these communities was through detours that are two to five times longer than the direct route [10]. In addition, residents of Hebron city- North of the West Bank, especially those living in the old city were required to take detours to get to the study clinic due to movement restrictions within the city. Gaining entrance to the remaining 15 communities was a challenge as they were all selected from “seam zone” areas. Most of these areas have been designated as closed military zones, which requires those aged 16 and above to apply for ‘permanent resident’ permits to continue living in their own homes. Entrance and exit of nonresidents requires special permits or coordination with the Crossing Point Administration (CPA) of the Israeli Ministry of Defense [10].

### Solution

Since trained fieldworkers were not allowed into these communities, the study team contacted community leaders who identified community members who were able to collect the data, and had a valid permit to enter and leave these communities. Two training sessions on standardized data collection were held for each fieldworker in villages neighboring their communities.

The UNRWA medical team was still required to visit each of these communities to collect physical measures, blood, and urine samples. Unlike fieldworkers, these teams could not be replaced by community members as the latter do not have the proper clinical training. No problems were anticipated for the medical team since UNRWA is a United Nations (UN) agency and its personnel have access to all parts of the West Bank. UNRWA’s operations team initially received approval to enter the seam zones from the Israeli District Civilian Liaison officer. Yet, on the first trip, UNRWA’s car was denied entry into the community and the team was informed that even UN personnel require permits to enter seam zone areas.

Based on previous experience with requesting permits from the Israeli military, and the delays and rejections received by fieldworkers, the study team decided to find an alternative way for the medical teams to enter these areas. The only way these teams could access these communities was to get to them from the Israeli side of the separation wall as there are no movement restrictions from that side once already in Israel. Only UNRWA employees living in Jerusalem (Center of West Bank) and holding certain IDs were allowed to enter Israel, this limited the number of teams that could complete the data collection in these communities. Organizing this further delayed fieldwork; the team was not expected to arrive to each community before 10:00 am due to the longer distance they had to travel as they all lived in the center of the West Bank, whereas a large number of the seam zone communities are located in the North and South.

In addition to challenges posed by movement restrictions, further difficulties were faced in finding locations within the community for the mobile clinics’ operations. Since seam zone areas are considered military zones, new construction as well as repairs of any buildings or infrastructure are restricted. Households are therefore very crowded and there is no space for public facilities including space for village councils, clinics, or even schools. In none seam zone communities, the mobile clinic for the PURE study were located in one of the rooms in the municipality or village council; in some cases it was also possible to use a clinic located in the community. Due to limited public space in seam zone communities working conditions in mobile clinics were sub-optimal. A different strategy was adopted depending on the circumstances in each community. The mobile clinic was set up in the community clinic if available. These “clinics” were poorly equipped and did not include any lab facilities, the team had to be fully equipped with material as basic as alcohol. The team also had to carry major equipment such as centrifuges to spin the blood samples. When a clinic was not present in the community, participants were asked to offer a room in their house to work from. Participants were generally cooperative and always provided space. The conditions of the space provided varied, some rooms did not have any electricity and the nurse had to keep the doors open to get light in.

## Considerations related to study setting

### Problem

Research in any setting requires knowledge of the local context. This has been previously cited as an important consideration to facilitate research and data collection [11], especially when data collection requires interaction with the general population. Gender of the fieldworker was important in the oPt. For example it was not acceptable for a male interviewer to approach females in the communities included in the study.

Other cultural considerations included respecting customs during the month of Ramadan as well as during olive picking season. People change their lifestyle, eating habits and social habits at these times of the year. It was important to monitor how these changes affected data collection. During Ramadan people eat just before sunrise so that they can postpone their next drink and meal till after sunset. This means that by the time they are ready to visit the mobile clinic for the PURE study they may not have fasted according to the study protocol (12 h). Many families in rural and urban communities depend on olive picking for a large portion of their household income. They either have their own trees to harvest, or they are hired by others with land to harvest their trees. Olives have to be harvested soon after they are ripe to prevent damage. Usually everyone in the household teams up to complete the harvest on time; people who are employed take time off work during this season. Everyone in the household leaves very early in the morning for olive picking and return late in the day. This delayed recruitment as households selected into the study were empty during the day.

### Solution

When possible, fieldworkers worked in teams of one male and one female for each community. Female fieldworkers were collecting data from females who mostly visited households during the day, because most females were homemakers. When encountering a household with a working female, the fieldworker was instructed to visit the household again in the afternoon. Male fieldworkers, who only interviewed males, were all instructed to make their visits in the afternoon to insure a more representative sample of working males.

There was a noticeable decline in response rate during the month of Ramadan and recruitment was paused in two communities until the month was over. This would not have been picked up if the research team was not closely monitoring the data collection process, and received daily updates from the field, thus compromising response rate. Ramadan also posed a challenge in communities where the mobile clinics had already started data collection as it was difficult for participants to complete 12 h of fasting. Working hours were changed in these mobile clinics to start working later in the day. Similarly, during olive picking season, recruitment and mobile clinics were interrupted in all rural communities until the harvest season was over.

## Conclusions

Some of the challenges faced during this study are similar to challenges raised by researchers in other low- and middle- income countries, such as cultural considerations and working in remote areas with limited resources [14]. Other challenges, such as access restrictions and working in a fragmented health care system, are specific to areas with chronic conflict. Our experience indicates that understanding the local context is very important in overcoming these challenges. We had anticipated most of these challenges and thus planned to overcome them.

A total of 1600 participants were recruited into the PURE study from the West Bank. The sample ensured representation of urban and rural communities, and accounted for individuals living in Palestinian refugee camps and seam zone areas, two settings unique to Palestinians. In order to ensure a full representation of the entire population of Palestine, it is important to recruit participants living in the Gaza Strip, and this is expected to lead to new challenges, given the siege on Gaza and the periodic attacks. Understanding the challenges and coming up with innovative ways to overcome these challenges is a step forward in increasing research from low- and middle- income countries.

This paper sheds light on a few unique challenges experienced during the data collection of a large epidemiology study in the oPt. We hope that this experience provides an impetus for other researchers and research projects to be conducted in conflict settings. Lessons learned could be useful for research among refugees from the current conflict in Syria and the rest of the Middle East.

## Abbreviations

CPA: Crossing Point Administration of the Israeli Ministry of Defense
MoH: Ministry of Health
NGOs: Nongovernmental Organizations
oPt: Occupied Palestinian Territory
PURE: Prospective Urban Rural Epidemiology
UN: United Nations
UNRWA: United Nations Relief and Works Agency for Palestine Refugees in the Near East
